# Sequelae and Quality of Life in Patients Living at Home 1 Year After a Stroke Managed in Stroke Units

**DOI:** 10.3389/fneur.2019.00907

**Published:** 2019-08-21

**Authors:** Sophie Broussy, Florence Saillour-Glenisson, B. García-Lorenzo, Francois Rouanet, Emilie Lesaine, Melanie Maugeais, Florence Aly, Bertrand Glize, Roger Salamon, Igor Sibon

**Affiliations:** ^1^Univ. Bordeaux, ISPED, Centre INSERM U1219-Bordeaux Population Health, Bordeaux, France; ^2^INSERM, ISPED, Centre INSERM U1219-Bordeaux Population Health, Bordeaux, France; ^3^CHU de Bordeaux, Pôle de Santé publique, Service d'Information Médicale, Bordeaux, France; ^4^Health Technology Assessment Unit, Hospital Clinic, University of Barcelona, Barcelona, Spain; ^5^Pôle des Neurosciences Cliniques, CHU Bordeaux, Bordeaux, France; ^6^Physical and Rehabilitation Medicine Unit, EA4136, Bordeaux University Hospital, University of Bordeaux, Bordeaux, France

**Keywords:** stroke, cognition, depressive disorder, fatigue, activities of daily living, social participation

## Abstract

**Introduction:** Knowledge about residual deficiencies and their consequences on daily life activities among stroke patients living at home 1-year after the initial event managed in stroke units is poor. This multi-dimensional study assessed the types of deficiencies, their frequency and the consequences that the specific stroke had upon the daily life of patients.

**Methods:** A cross-sectional survey, assessing, using standardized scales, 1 year post-stroke disabilities, limitations of activities, participation and quality of life, was carried out by telephone interview and by mail in a sample of stroke patients who returned home after having been initially managed in a stroke unit.

**Results:** A total of 161 patients were included (142 able to answer the interview on their own; 19 needing a care-giver). Amongst a sub-group of the patients interviewed, 55.4% (95% Confidence Interval [47.1–63.7]) complained about pain and 60.0% (95% CI [51.4–68.6]) complained of fatigue; about 25% presented neuropsychological or neuropsychiatric disability. Whilst 87.3% (95% CI [81.7–92.9]) were independent for daily life activities, participation in every domains and quality of life scores, mainly in daily activity, pain, and anxiety subscales, were low.

**Conclusion:** Despite a good 1-year post-stroke functional outcome, non-motor disabling symptoms are frequent amongst patients returned home and able to be interviewed, contributing to a low level of participation and a poor quality of life. Rehabilitation strategies focused on participation should be developed to break the vicious circle of social isolation and improve quality of life.

## Introduction

Stroke is the leading cause of acquired physical disability in adults worldwide. Although stroke units have dramatically improved post-stroke functional outcome and reduce post-stroke mortality (1) by focusing on acute stroke management, they often failed to consider stroke as a chronic disorder with potential delayed neuropsychological and emotional consequences. Indeed, while motor impairment, spasticity or aphasia are easily recognized complications, other deficiencies, such as cognitive impairment (2), depression (3), or fatigue (4) are also frequently reported but under-evaluated and poorly managed amongst stroke survivors. These so-called “invisible” deficiencies are thought to contribute to reduced ability to participate in daily life activities and also impaired quality of life (5). Furthermore, while most of patients able to return at home following stroke managed in stroke units are supposed to have a good outcome, they may suffer from a lack of monitoring.

Residual deficiencies and their consequences upon the daily life activities have mainly been assessed in large and representative populations of stroke patients (6). However, little information is available about post-stroke sequelae in the population of patients living at home and having been managed in stroke units.

Moreover, most of studies assessing post-stroke sequelae are focused on a small spectrum of sequelae; and present a high variability of results due to heterogeneity of populations, study designs, diagnostic scores and stroke types (4, 7, 8).

To address this lack of information, we adopted a multi-dimensional approach to assess the frequency and type of deficiencies with a focus on their daily-life consequences in a cohort of patients who are living at home 1 year after stroke having managed in stroke units.

## Methods

A cross-sectional survey of 1-year post-stroke deficiencies, limitations in activities, participation and quality of life was conducted in a population of patients, included in the Aquitaine Observatory of Stroke (ObA2), from October 1st to December 4th 2013 with a stroke managed in one of the stroke units participating to ObA2.

ObA2 is a regional cohort of patients with a recent stroke, diagnosed and managed in the short-stay services of the hospitals in the Aquitaine region, Southwestern France. ObA2 objectives are to (1) describe the management of stroke (practices, delays, referral of patients, etc.) prior to hospitalization, during hospitalization and in the post-hospital phase, (2) describe the population of stroke patients in socio-demographic and clinical terms, (3) monitor the population of these patients in terms of: occurrence of complications during the stay, mortality at 1 year, disability at 1 year. ObA2 is the only stroke cohort in France covering a whole region, allowing the inclusion of a large and heterogenic panel of stroke patients.

The Oba2 inclusion criteria are: (i) aged over 18 years (ii) living in metropolitan France; (iii) suffering from a recent stroke whose diagnosis is confirmed by a neuro-vascular physician; (iv) stroke managed in one of the participating hospitals of the Aquitaine region with more than 30 strokes per year; (v) giving consent to participate. The study sample included patients of the study population alive at 1-year after a stroke managed in a stroke unit, living at home at 1 year post-stroke, with an available phone number at ObA2 admission and willing to participate. According to French regulations applicable at the time of research (9), this study was exempted from examination by an Ethics Committee in France and did not require written or verbal informed consent to participate. Oba2 cohort had received authorization from the French Commission nationale de l'informatique et des libertés (authorization n°911201). According to regulations, patients received oral and written information at Oba2 inclusion about the project, its objectives and data management process and about their refusal rights and process. Moreover, the study has been presented to the local institutional review board of the Bordeaux University Hospital.

Demographic data, stroke characteristics at admission (type of stroke, gravity of stroke assessed by the National Institute of Health Stroke Score-NIHSS), modified Rankin scale (mRS) at discharge, complication during acute stay, rehabilitation during acute stay, length of stay and admission to rehabilitation service after discharge or during follow-up were recorded at Oba2 inclusion using medical records. A selection of scales measuring each of the sequelae explored were selected, based on elements of feasibility, frequency of use in the exploration of the dimension considered and metrological performance (validity and reproducibility). The mode of collection of sequelae has been adapted according to the validated mode of administration for each of the scales.

At 1-year, two types of evaluations were performed, an initial interview conducted by telephone followed by self-assessment questionnaires which were sent by mail. Patients were asked to return their responses to the questionnaires using a prepaid envelope.

After 4 attempts, patients that could not be reached by phone were qualified as non-responders. The non-responders vital status was carried out in the local death registry. Those for whom vital status could not be retrieved 1 year post-stroke were considered as lost-to-follow-up. When patients were unable to answer questions on their own due to aphasia or severe cognitive impairment, caregivers were asked to answer the phone interview and complete the questionnaires on the patients' behalf.

Data recorded during the phone interview were: socio-economic characteristics (living alone, ability to drive), cognition and pain using the French Telephone Interview for Cognitive Status Modified (F-TICS-m) (10) and the simple verbal scale (EVS) (11) questionnaires, dysphagia, sphincteric and genito-sexual disorders using closed-ended question “yes/no,” and functional disability using the mRS (12). Data recorded using self-assessment questionnaires were: depression and anxiety using the hospital anxiety and depression scale (HADS) (13), fatigue using the Fatigue Severity Scale (FSS) (14), functional outcome using the Barthel Index (BI) (15). Moreover, we explored several categories of participation according to the ICF model (16) and focused on Domestic Life, Economic/social productivity, Interpersonal Interactions and Relationships using the Community Integration Questionnaire (CIQ) which is composed by a global score and three sub-scores (integration at home, social integration, productivity) (17). The higher these sub-scores are, the higher the participation in specific categories is. Finally, because limitations in activities or participations do not necessarily impact the quality of life, we explored the latter using the EUROQOL (EQ5D-3L) (18). An EQ-5D utility score for each patient 1-year post-stroke was calculated using information from the EQ5D-3L and a validated algorithm to calculate index values for the EQ-5D instrument (19). The mean value of the EQ-5D utility score and the standard error associated was calculated for the patients' sample and subgroups (19).

Three groups were considered: the entire study sample and 2 sub-groups of patients (interviewed and unable to answer the phone interview). Analyses presented in the article are focused in the sub-group of interviewed patients. Wilcoxon Mann-Whitney test was used to compare continuous variables. Chi-square test with or without Yates correction and Fisher's exact were used to compare categorical variables. *P* < 0.05 was considered statistically significant. Spearman correlation tests were performed to study the relationship between F-TICS-m, EVS, HADS, FSS, CIQ, or EQ5D-3L scores and either mRS or BI scores.

The data that support the findings of this study are available from the corresponding author upon reasonable request.

## Results

**Figure 1:**
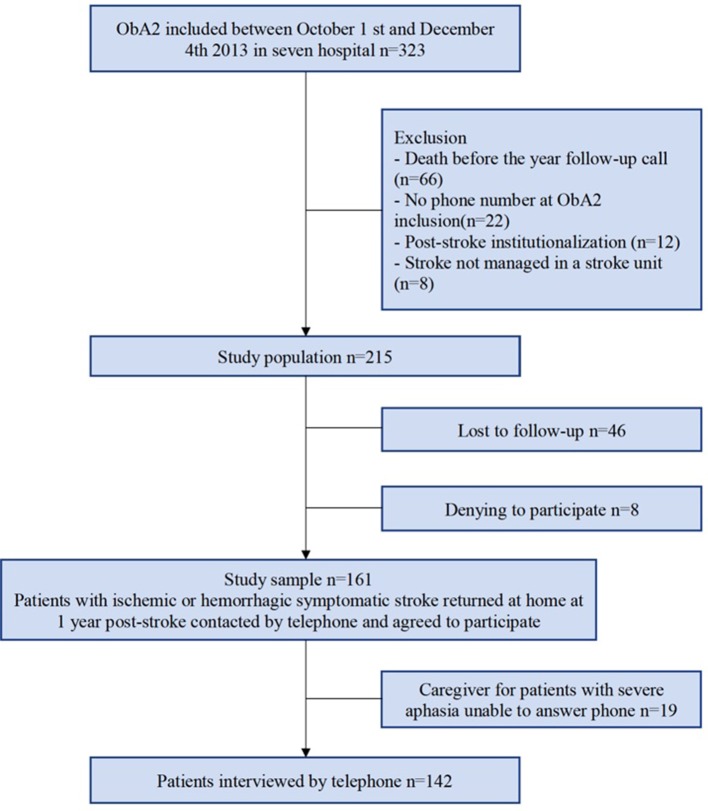
Study flow-chart.

Of the 215 patients of the study population, 161 were included in the final study sample, amongst whom 142 were able to answer on their own and 19 needed a caregiver to answer the phone interview (Figure 1, Table 1). A total of 134 patients returned the self-assessment questionnaires.

**Table 1:** Socio-demographic and clinical characteristics: study sample, patients lost-to-follow-up.

			**Study sample**				
	**Patients lost to follow-up** ***n*** **=** **46**		**All** ***n*** **=** **161**		**Patients own responses** ***n*** **=** **142**		P^*^
	***n***	**%**	***n***	**%**	***n***	**%**	
Sex (Female)	19	41	61	38	54	38	NS
Age over 75 years (in years)	21	46	66	41	59	42	NS
Level of education (Below or equal to the baccalaureate)			137	85	120	85	
History of cardiovascular risk factors	40	87	141^‡^	88	125^§^	88	NS
Stroke	10	22	21	13	19	13	
Transient ischemic attack	2	4	7	4	6	4	
Atrial fibrillation	5	11	23	14	17	12	
Diabetes	6	13	26	16	21	15	
Hypertension	31	67	98	61	87	61	
Dyslipidemia	19	41	56	35	52	37	
Smoke	12	26	52	32	46	32	
Presence of handicap before stroke (mRS>1)	9^||^	19	15^#^	9	12^††^	8	NS
NIHSS score at admission							0.0006
≤5	17^‡‡^	37	100^§§^	62	93	65	
[6-13]	18	39	30	19	26	18	
>13	9	19	18	11	12	8	
Ischemic stroke	37	80	141	88	122	86	NS
Complication during acute stay^**^	33	72	85^||||^	56	74^##^	55	NS
Rehabilitation during acute stay	28^††††^	74	87^‡‡‡^	67	74^§§§^	65	NS
Modified Rankin score ≤ 2 at discharge	25	54	112	70	106	75	0.0100
Length of stay (>8 days)	29	59	75^||||||^	47	61	43	0.0498
Orientation to rehabilitation services at discharge	20	41	55	34	49	34	NS
Admission to rehabilitation services during 1 year after stroke episode	3	6	11	7	10	7	NS
Stroke recurrence during follow-up			15	9	13	9	
Living alone 1 year after stroke			36	22	33	23	

* *Comparison between group of patients interviewed (study sample) and group of patients lost to-follow-up*.

** *Complication during acute stay: neurological complication, infectious complication, cardio-vascular complication, other*.

‡ *2 data missing for cardiovascular risk factors*.

§ *2 data missing for cardiovascular risk factors*.

|| *4 data missing for handicap before stroke*.

# *22 data missing for handicap before stroke*.

†† *20 data missing for handicap before stroke*.

‡‡ *2 data missing for NIHSS score at admission*.

§§ *13 data missing for NIHSS score at admission*.

|||| *9 data missing for complication*.

## *8 data missing for complication*.

†††† *8 data missing for rehabilitation*.

‡‡‡ *31 data missing for rehabilitation*.

§§§ *29 data missing for rehabilitation*.

|||||| *1 data missing for length of stay*.

Mean age of the subgroup of the 142 interviewed patients was of 69 years (Standard Deviation-SD: ± 14.0), the male/female ratio was of 1.6 and 86.0% of strokes were ischemic. The mean NIHSS score at admission was of 4.7 (SD: ± 5.5). Fifty six percent of the patients presented complication during acute in-hospital stay (neurological complication—7%, infectious complication-−11%, cardiovascular complication—4%). At hospital discharge, 74.6% of study sample patients had a mRS ≤ 2 and 62.7% returned directly to home. A total of 55% of patients received early rehabilitation during the acute stay, 34% were admitted directly to rehabilitation at discharge and 7% during follow-up.

The comparison of the socio-demographic characteristics and the clinical history between the sub-sample of interviewed patients and the group of patients lost-to-follow-up showed no statistical differences. However, a higher proportion of patients lost-to-follow-up had a length of stay >8 days (*p* = 0.049), a severe NIHSS score at admission (>13), (*p* = 0.0006) and a lower proportion of these patients had a mRS ≤ 2 (*p* = 0.01). The group of patient lost-to-follow-up seemed to have presented a more severe stroke with a less positive prognostic than the interviewed patients.

Overall, 21.8% (95% Confidence Interval - CI [15.0–28.6]) of the subgroup of interviewed patients had a cognitive impairment and 60.0% (95% CI [51.4–68.6]) reported high fatigue (FSS≥ 5.5/7) (Table 2). About one quarter of this group of patients reported anxiety and depressive disorders (HADS≥11/14) and 55.4% (95% CI [47.1–63.7]) complained about pain. More than two-thirds of the interviewed patients showed good functional outcome: 70.4% (95% CI [62.9–77.9]) reported a mRS ≤ 2 and 87.3% (95% CI [81.7–92.9]) were assessed as independent enough to live at home (BI≥60). Evaluation of participation demonstrated low levels of participation (CIQ global score: mean 13.5/29; SD ± 6.6; 95% CI [12.3–14.6]), mainly in the domains of home integration (CIQ-Home integration: mean 3.8/12; SD ± 2.9) and productivity (CIQ-Productivity: mean 3.07; SD ± 2.3). Quality of life measured as the mean EQ-5D utility score is 0.67 (SD ± 0.03; Table 2).

**Table 2:** Frequency of 1 year post-stroke sequelae and quality of life of study sample.

		**All** ***n*** **=** **161**			**Sequelae reported by patients** ***n*** **=** **142**			**Sequelae reported by caregivers*****n*** **=** **19**	
		***n***	**%**	**IC 95%**	***n***	**%**	**IC 95%**	***n***	**%**
Disabilities									
Declarative	Dysphagia	16	9.9	[5.3–14.6%]	13	9.1	[4.4–13.8%]	3	15.7
Declarative	Sphincteric disorders	42	26.0	[19.3–32.9%]	38	26.8	[19.5–34.1%]	4	21.0
Declarative	Genito-sexual disorders	32	19.9	[13.7–26.0%]	30	21.1	[14.4–27.8%]	2	10.5
TICS	Cognitive impairment (TICS^*^ <24/43) (*n* = 142)	31	21.8	[17.1–30.9%]	31	21.8	[15.0–28.6%]		
HADS	Anxiety disorders (HADS^**^≥11/14) (*n* = 146)	35	24.0	[17.1–30.9%]	32	24.1	[16.8–31.4%]	3	23.1
	Depressive disorders (HADS ≥11/14) (*n* = 146)	37	25.3	[18.3–32.4%]	30	22.5	[15.4–29.6%]	7	53.8
FSS	High fatigue (FSS^***^≥5.5/7) (*n* = 141)	84	59.6	[51.5–67.7%]	75	60.0	[51.4–68.6%]	9	56.3
Pain scale	Pain (*n* = 154)	87	56.5	[48.7–64.3%]	77	55.4	[47.1–63.7%]	10	66.7
Limitations of activities									
mRS	No handicap or low handicap (mRS^‡^ ≤ 2)	107	66.5	[59.2–73.8%]	100	70.4	[62.9–77.9%]	7	36.8
Barthel Index	Autonomy to return home (BI^||^≥60) (*n* = 148)	124	83.8	[77.9–89.7%]	117	87.3	[81.7–92.9%]	7	50.0
Driving before stroke and resumption after stroke		87	54.0		81	57.0		6	31.6
		**Mean**	**SD**	**IC 95%**	**Mean**	**SD**	**IC 95%**	**Mean**	**SD**
Quality of life									
EQ5D-3L^δ^	Utility score (*n* = 142)	0.63	0.37	[0.56–0.69]	0.63	0.37	[0.56–0.69]	0.24	0.48
Participation									
CIQ^§^	Global score (/29) (*n* = 143)	13.0	6.8	[11.9–14.1]	13.5	6.6	[12.3–14.6]	12.6	6.6
	Home integration (/12) (*n* = 146)	3.6	2.9	[3.1–4.0]	3.8	2.9	[3.3–4.3]	1.0	2.0
	Social integration (/10) (*n* = 144)	6.5	2.9	[5.9–6.9]	6.6	2.8	[6.1–7.1]	4.2	3.1
	Productivity (/7) (*n* = 145)	2.9	2.2	[2.4–3.2]	3.0	2.3	[2.6–3.4]	1.8	1.9

* Telephone Interview for Cognitive Status;

** Hospital anxiety and depression scale;

*** Fatigue Severity Scale;

‡ Modified Ranking scale;

|| Barthel Index;

§ Community Questionnaire;

δ *Health Organization Quality of Life-EUROQOL (the utility values range from 0 for death to 1 for perfect health)*.

The correlation analyses showed that the higher the limitations of activities were, the lower participation (CIQ-Home integration: *r* = −0.50. *p* < 0.001; CIQ-Social integration: *r* = −0.57, *p* < 0.001; CIQ-Productivity: *r* = −0.49, *p* < 0.0001), and the higher the limitations of activities were, the lower the quality of life (mRS: *r* = −0.67; BI: *r* = 0.72; Table 3).

**Table 3:** Spearman correlation coefficients between F-TICS-m, EVS, HADS, FSS, or EQ5D-3L scores and either mRS or BI scores.

	**Limitations activities**		
		**Barthel (*n* = 134)**	**Rankin (*n* = 142)**
**Disabilities**			
Cognitive impairment	TICS (*n* = 142)	0.43^*^	−0.58^*^
Anxiety disorders	HADS (*n* = 133)	−0.24^*^	0.19^*^
Depressive disorders	HADS (*n* = 133)	−0.49^*^	0.42^*^
Fatigue	FSS (*n* = 125)	−0.46^*^	0.31^*^
Pain	EVS (*n* = 139)	−0.35^*^	0.41^*^
**Participation**			
Global score	CIQ (*n* = 130)	0.57^*^	−0.60^*^
Home integration	CIQ (*n* = 133)	0.44^*^	−0.50^*^
Social integration	CIQ (*n* = 131)	0.49^*^	−0.57^*^
Productivity	CIQ (*n* = 132)	0.55^*^	−0.49^*^
**Quality of life**			
EQ5D-3L	*n* = 128	0.72^*^	−0.67^*^

*Group of interviewed patients*.

* *Univariate Spearman's rank correlation analysis showed a significant correlation between two variables*.

## Discussion

This study providing original data on the outcome of a specific population of stroke patients living at home after stroke managed in a stroke unit suggests that (i) pain and fatigue represents the main persistent symptoms while cognitive and neuropsychiatric disabilities are observed in about 20% of the population, (ii) despite an overall good functional outcome, patients who are independent in their daily-life activities have multidomain impairment in participation and a lower level of quality of life scores compared to the general population at the same age range in France (0.81) (20).

The frequency of pain and fatigue as well as cognitive and neuropsychiatric disabilities observed in our selected population with a good functional outcome is very similar to what is usually reported in the global stroke population (21). This result suggests that the rate of “invisible handicap” is persistent across patients independently from stroke severity. Interestingly, the level of home and social integration as well as productivity and quality of life remains very low in this population suggesting strong participation restrictions and daily life consequences despite a good functional outcome. The predominance of restriction participation in the field of home integration might reflect the difficulties of stroke patients to face their new physical condition in their own environment with overprotective relatives. Moreover, the strong correlation between objective and subjective measurements of deficiencies and limitations of activities shows the critical consequences of these sequelae on post-stroke quality of life.

Specific rehabilitation and occupational therapy in the patients' living place might improve the home integration of these patients. Impaired cognition, and the well-known difficulty to perform dual-task in stroke patients (22) could also contribute to this restriction of participation suggesting that cognitive rehabilitation is mandatory to improve the probability for patients to reach a level of participation similar that which they had prior to the stroke. The difficulties of reintegration at home and in society could in turn increase anxiety and depression, fatigue, and lead to a vicious circle of social isolation with its impact upon patients' quality of life which must be broken (23).

Interestingly the high rate of invisible deficiencies observed in this study was observed despite an initial acute management of these patients in a stroke unit that should have ensured optimal outcomes. Despite real improvement in the access to stroke units in Europe, about 4% to 50% of stroke patients are not admitted to stroke units; the rate of invisible handicap in this population might be even higher than in the present cohort, and highlight the need to build dedicated workflow for all stroke patients to improve their outcome.

However, the results have to be interpreted cautiously due to several limitations represented by the specificity of the French healthcare system and cultural factors, the low number of patients included and their clinical characteristics. Indeed, the low level of severity at admission (62–65% with the NIHSS score at admission ≤5) and good functional outcome at hospital discharge (74.6% had mRS ≤2) suggest that the present results cannot be generalized to the whole stroke population admitted in stroke units. Moreover, only 66% of the patients responded on their own to the interview which might represent a significant bias to interpret the results. Similarly to those who were lost to follow-up and those who denied to participate we can assume that those who were unable to answer on their own had an even worse functional outcome, suggesting that the present study underestimate the true daily-life consequences of stroke in patients with minor to mild stroke.

Because the improvement of acute stroke treatments now offers considerable reduction in the severity of physical disability, there is an urgent need to develop more systematic detection and management of the remaining invisible deficiencies that contribute to persistent restriction of participation in daily-life.

## Data Availability

The datasets generated for this study are available on request to the corresponding author.

## Ethics Statement

The study was approved by the local ethics committee and the ObA2 cohort had received CNIL authorization (n°911201). All patients received individual written information.

## Author Contributions

SB, FS-G, BG-L, FR, EL, MM, FA, BG, RS and IS contributed to the study design, data collection and manuscript writing. SB and BG-L performed statistical analyses.

### Conflict of Interest Statement

The authors declare that the research was conducted in the absence of any commercial or financial relationships that could be construed as a potential conflict of interest.
